# Platelet signaling - blood's great balancing act

**DOI:** 10.18632/oncotarget.5122

**Published:** 2015-08-07

**Authors:** Wolfgang Bergmeier, Lucia Stefanini

**Affiliations:** Department of Biochemistry and Biophysics, McAllister Heart Institute, University of North Carolina at Chapel Hill, Chapel Hill, NC, USA

**Keywords:** GTPase, platelets, thrombosis, balance, signaling

Platelets play an essential role in the body's ability to respond to vascular injury (hemostasis). To form a hemostatic plug and prevent bleeding from the damaged blood vessel, these cells need to be equipped with a powerful signaling machinery that allows them to respond to trace amounts of agonists produced at injury sites, and to rapidly transition from an anti-adhesive/patrolling state to an adhesive state (integrin inside-out activation) required for plug formation. However, this highly sensitive signaling machinery can also pose a significant risk, as premature platelet activation can lead to thrombocytopenia (impaired platelet homeostasis) and/or thrombosis. Thus, negative feedback within the signaling machinery is crucial to balancing sensitivity and speed.

Circulating platelets are also important in various aspects of cancer [1]. They support tumor growth by providing necessary growth and angiogenic factors. They facilitate tumor metastasis by multiple mechanisms, including protection of tumor cells from immune surveillance while in circulation and adherence to the microvasculature at sites of extravasation. To engage platelets for these tasks, tumor cells have developed ways to activate and aggregate these cells. Consistent with these findings, cancer has emerged as a major risk factor for thrombotic disease, and a high peripheral platelet count correlates with poor survival in various cancers [1]. A better understanding of the signaling machinery that regulates platelet reactivity is required to determine who is at an increased risk for thrombotic disease, both in the absence and presence of cancer.

We and others identified the small GTPase RAP1 as a critical regulator of platelet adhesiveness [2]. RAP1 serves as a cellular switch that cycles between a GDP-bound “off-state” and a GTP-bound “on-state”, the latter being required for integrin activation. In platelets, RAP1 activation strongly depends on the guanine nucleotide exchange factor, CalDAG-GEFI (RasGRP2). CalDAG-GEFI activity is regulated by a pair of highly sensitive EF hand domains (K_D_~80nM) [3, 4]. These regulatory domains provide remarkable sensitivity towards minor changes in the cytoplasmic calcium concentration, which in resting platelets was measured at ~20-50nM. Consistent with this model, platelets lacking functional CalDAG-GEFI show markedly impaired sensitivity towards threshold concentrations of agonists, both *in vitro* and *in vivo*. In addition to sensitivity, CalDAG-GEFI is also critical for the near-immediate activation of RAP1, triggered by rapid calcium mobilization from stores in the endoplasmic reticulum. Both RAP1 activation and integrin inside-out activation are delayed in platelets from mice and humans lacking functional CalDAG-GEFI [3, 5, 6].

As outlined above, a highly sensitive signaling machinery needs to be tightly controlled to prevent premature platelet activation. In recent work, we identified the RAP-GAP, RASA3 (GAP1^IP4bp^, R-RAS-GAP) as a critical negative regulator of RAP1 signaling in platelets [7]. RASA3 is perfectly positioned to prevent premature integrin activation as it is anchored to the plasma membrane of resting platelets by a unique PH/Btk domain. Deletion of functional RASA3 in mice led to premature activation and clearance of platelets from circulation. Concomitant deletion of CalDAG-GEFI reversed this phenotype in *Rasa3* mutant mice, strongly suggesting that RASA3 is critical to restrain the highly sensitive CalDAG-GEFI/RAP1 signaling pathway. Thus, one can imagine RASA3 as a “hand brake”, which is pulled in circulating platelets to maintain the cells in a resting state. At sites of vascular injury, however, this brake would impair RAP1 activation and firm platelet adhesion and thus needs to be released to allow for hemostatic plug formation. Our studies strongly suggest that RASA3 inactivation is dependent on signaling by phosphoinositide 3 kinase (PI3K), downstream of the platelet receptor for ADP, P2Y12. More specifically, we observed that *Rasa3* mutant platelets did not require feedback signaling via ADP and P2Y12, and that RAP1-dependent integrin activation in these cells was insensitive to inhibitors of P2Y12 or PI3K. Consistent with this data, platelet adhesion at sites of injury in *Rasa3* mutant mice was insensitive to inhibitors of P2Y12.

**Figure 1:**
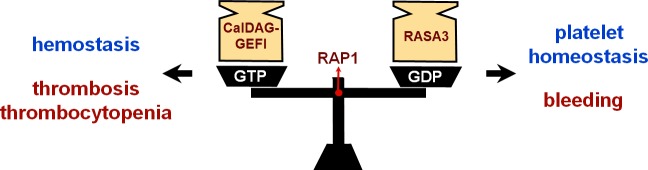
The antagonistic balance between the RAP GTPase regulators, CalDAG-GEFI and RASA3, controls platelet adhesiveness both in circulation and at sites of vascular injury

Together, these studies suggest that the antagonistic balance between CalDAG-GEFI and RASA3 signaling is critical for the fine-tuning of platelet adhesiveness, both in the circulation and at sites of vascular injury. Genetic and environmental factors that shift the balance towards RAP1 activation may lead to thrombocytopenia and thrombosis, while those that lead to impaired RAP1 activation may cause bleeding in patients (Figure 1). Future efforts should be directed towards the testing of this hypothesis in humans, studies that will greatly benefit from the development of novel assays that monitor the expression and activity of CalDAG-GEFI, RASA3, and RAP1. The long-term benefit of this work could be a more personalized approach to thrombosis prevention, which is based on inter-individual differences in the regulation of this important platelet signaling pathway.
